# Targeting Hsp90 in Cancer for 25 Years: Failure of Previous Clinical Trials and New Hope for Future Therapeutics

**DOI:** 10.3390/cells14241989

**Published:** 2025-12-15

**Authors:** Mei Chen, Cheng Chang, Kathleen L. Miao, David T. Woodley, Wei Li

**Affiliations:** Department of Dermatology, The Keck School of Medicine, University of Southern California, Los Angeles, CA 90033, USA; chan296@usc.edu (C.C.); klmiao@usc.edu (K.L.M.); dwoodley@usc.edu (D.T.W.)

**Keywords:** intracellular Hsp90, extracellular Hsp90, old and new generations of inhibitor therapeutics, cancer clinical trials, paradigm shift

## Abstract

All previous IND (investigational new drug) applications to US FDA for launching clinical trials with Hsp90 ATP-binding inhibitors only provided a partial, if not misleading, account of the inhibitors’ actual MOA (mechanism of action). Since 2004, studies have repeatedly shown a previously unanticipated “extra effect” of these inhibitors, but it has been incomprehensively ignored by the Hsp90 community. Membrane-impermeable, otherwise structurally identical, ATP-binding Hsp90 inhibitors show robust inhibition of tumor cell invasion in vitro and metastasis in vivo. Based on this new finding, the reported outcomes of around 90 monotherapy clinical trials with Hsp90 ATP-binding inhibitors since 1999 were actually a combined effect of targeting both intracellular Hsp90 chaperone and extracellular Hsp90 (eHsp90) non-chaperone functions by the inhibitors. A critical unanswered question remains: which form of the dual inhibitions caused the observed toxicity in humans that led to the spectacular failure of the trials and which underlies the limited efficacy that might be the real reason for the only approval of the orally administered ATP-binding inhibitor, Pimitespib (TAS-116), in 2022 by Japan? We suggest that addressing this question could prompt a paradigm shift in the design of next-generation anti-Hsp90 cancer therapeutics.

## 1. Introduction

Protein quality control and the maintenance of proteome homeostasis—collectively referred to as proteostasis—in the cytosol of all cells are essential for sustaining cellular and organismal health. Disruption or intrinsic malfunction of this evolutionarily conserved housekeeping system, such as pathological stress or age-related decline, is associated with a wide range of disorders, including autoimmune and neurodegenerative diseases, in humans [1]. Proteostasis is maintained by an extensive network of molecular chaperones, including Hsp60, Hsp70, Hsp90, Hsp104, and the small heat shock proteins. The so-called “molecular chaperones” are historically defined as proteins that interact with, stabilize, or assist those polypeptides that need the chaperones to attain their functionally active conformations via cofactor-regulated binding and release cycles, typically in an ATP-dependent manner. When the nascent polypeptides from ribosomes have achieved their functional forms, these chaperones depart and release the mature structure of the client proteins. A common recognition feature among the client polypeptides is likely the presence of exposed hydrophobic amino acid side chains characteristic of non-native, nascent, or denatured protein states [2,3].

Among the chaperone proteins, Hsp90 is involved in the late stage of protein folding—following Hsp70—to protect nascent polypeptides emerging from the ribosomes from aggregation, which could lead cytotoxicity and death. In fact, there has been little direct evidence that Hsp90 is required to maintain binding to already fully folded and mature proteins under steady-state conditions. For instance, co-immunoprecipitation (co-IP), one of the widely used techniques to visualize protein–protein interactions from cells, would not be able to distinguish the actual nature of the pulled-down proteins between their nascent forms and mature forms, let alone the even less specific mass spectrometry (MS) analysis. Given Hsp90’s critical housekeeping role in proteostasis, Hartl has long cautioned “indiscriminate and global inhibition of Hsp90 would disrupt the proteostasis machinery, leading to toxicity before therapeutic benefit in humans”. Instead, he proposed that therapeutic strategies should aim to selectively inhibit specific aspects of the Hsp90 function—ideally those critical for tumor cell survival but dispensable in normal cells [4]. However, these cancer-specific vulnerabilities, if there are any, have never been substantiated.

## 2. An Extended Hsp90 Chaperone Theory

Since the mid-1980s, however, the definition of Hsp90’s “chaperone function” has been broadened—perhaps more accurately, “stretched”—to encompass presumed far greater responsibilities than the originally defined one. More noticeably, this expanded concept has since become the scientific foundation for anti-Hsp90 cancer clinical trials. Based largely on a handful of in vitro studies and non-definitive data of experiments, the traditional view of protecting nascent polypeptide chains and maintaining proteostasis was boldly extended into the now widely accepted theory that Hsp90 acts as a lifelong partner to hundreds of essential regulatory proteins or oncogenic mutants by maintaining the proteins’ stability throughout their lifespans. A key point in this extended theory is the assertion that dissociation of Hsp90 from any of its clients would inevitably lead to misfolding and subsequent degradation of the clients [5]. Classic examples include the Hsp90–steroid receptor complex during receptor-mediated gene expression [6], the oncogenic v-Src tyrosine kinase [7], and EGFR family proteins [8]. Based on the principles of protein chemistry, there is no reason for fully folded, mature, and undamaged proteins to expose hydrophobic regions to the aqueous cytosol under physiological conditions. In addition, it is hard to believe that carrying a 90 kda protein on its back does not sterically interfere with the 60 kda Src function. To date, no specific “epitope” or conserved recognition sequence has ever been identified among the so-claimed ~400 putative Hsp90 client proteins—an anomaly in the context of established biochemical principles for protein–protein interactions. To account for this, some have proposed that the extended surface of Hsp90 presents a mosaic of hydrophobic, hydrophilic, and charged patches, enabling diverse low-affinity interactions with different clients [9,10]. Any new theory with such a high level of non-neglectable caveats should have been more thoroughly scrutinized prior to serving as the foundation for clinical trials. Unfortunately, the practice forbidden by the ancient scholastic philosophy of Chinese, “人云亦云” (meaning “to follow the crowd”), has dominated the field over the past few decades.

Much of the evidence for the extended chaperone theory arises from two experimental approaches: antibody co-IP and MS analysis, as previously mentioned. However, these two methods of detection do not have the level of resolution to distinguish between interactions of Hsp90 with either a mature protein or its nascent polypeptide emerging from the ribosomes. A major caveat lies in the stoichiometry of the experimental data: the quantity of co-immunoprecipitated client proteins often deviates markedly from expected protein–protein interaction ratios, and these discrepancies could not be fully explained by differences in antibody affinity. For example, while anti-Hsp90 immunoprecipitation followed by Western blotting with an anti-client antibody might suggest that only a small fraction of the Hsp90 pool associates with a given client, the reverse experiment—precipitating with an anti-client antibody and probing for Hsp90—should yield a comparable Hsp90: client ratio if the central tenets of the extended theory were correct. More notably, unlike many other standard protein–protein interaction studies, few previous investigations have utilized recombinant proteins to define the ratio of interaction, let alone identifying the binding epitope and quantifying the binding affinity. These deficiencies left a critical part of the extended Hsp90 chaperone model unverified.

## 3. A Further Extended Theory of the Hsp90 Chaperone Machine

Pushing the envelope even further to support the rationale for anti-Hsp90 cancer drug development, Chiosis and Neckers proposed an “activated state” of the Hsp90 complex in cancer cells versus a “latent state” in normal cells [11]. A study from Chiosis’ group designated the activated state in tumor cells as “epichaperome” [12]. However, this hypothesis appears to contradict the well-established role of Hsp90 in chaperoning nascent peptide folding—a process that occurs continuously in all cell types in human body. If Hsp90’s primary function is to maintain cellular proteostasis and if tumor cells overexpress Hsp90 to achieve higher protective capacity, it is hard to comprehend how Hsp90 inhibitors harm tumor cells preferentially over normal cells. No satisfactory explanation has ever been provided for the persistent disconnect between in vitro reports of “hundreds to thousands fold selectivity for tumor cells” or “no toxicity to normal cells” and the consistent lack of clinical efficacy observed in cancer patients. For example, Kamal and colleagues reported ~100-fold higher affinity of tumor cells than a different type of normal cells for binding to the Hsp90 inhibitor 17-AAG. However, these experiments were performed using broken cell extracts rather than intact cells, raising concerns about physiological relevance [13]. Subsequent attempts to replicate these findings failed to confirm the existence of an exclusive high-affinity Hsp90 complex in cancer cells. Barrott and Haystead demonstrated that the purported high-affinity complex was an artifact arising from nonspecific binding to the affinity resin [14]. Ultimately, the repeated failures of Hsp90 ATP-binding inhibitor cancer clinical trials (>180) since 1999 cannot be adequately explained by increasingly speculative arguments invoking “oncogenic signaling”, “unique post-translational modifications of Hsp90”, “distinct co-chaperones”, or similar tumor-specific features. These explanations remain unsubstantiated in the context of intact cellular systems and clinical outcomes.

## 4. The “Hsp90 Inhibitor Binding–Client Protein Degradation” Foundation for Cancer Clinical Trials Applies Only to Limited Tumor Cells

The widely accepted paradigm—Hsp90 inhibitor binding leading to dissociation and subsequent degradation of client proteins—appears to hold true only for a subset of tumor cell types. A fundamental limitation of this model is its inability to distinguish whether the degraded client proteins are mature, functional molecules or immature, nascent forms of polypeptides still undergoing folding and maturation following their synthesis by ribosomes. Early studies by Neckers and colleagues [7,15] revealed a puzzling feature: even for highly sensitive Hsp90 clients such as members of the EGFR family, inhibitor-induced dissociation and degradation required hours to days of continuous drug exposure. This is inconsistent with the rapid turnover expected for signaling proteins; for example, the half-life of cyclin D1 in normal cells is only 20–30 min. A specific challenging finding to the paradigm emerged from a recent study by Tang and colleagues, surprisingly few of such comparative studies that had previously been conducted to verify the paradigm prior to launch of clinical trials. Tang et al. examined the degradation patterns of client proteins from the mitogenic signaling pathway after standardized treatment with 100 nM 17-DMAG for 48 h among eight randomly selected cancer cell lines. They found that in five of the eight cell lines, client protein responses deviated from the expected degradation pattern. MDA-MB-231 breast cancer cells were the only line in which all five selected clients adhered strictly to the paradigm. Even in another triple-negative breast cancer line, MDA-MB-468, the same set of clients responded differently from MDA-MB-231 cells. These heterogeneous responses were unrelated to the total Hsp90 levels in each cell line. Similar variability had been observed previously in single-cell line studies by other independent groups [16]. Although possible explanations include post-translational modifications and complex oncogenic signaling networks, the precise causes remain speculative. Regardless, this heterogeneity poses a serious challenge to the central narrative that has guided Hsp90 inhibitor clinical trial design since 1999. Now we all know the outcomes of around 90 monotherapy trials—not a single FDA approval. Regarding the orally administered Pimitespib (TAS-116, Tablets 40 mg) by Taiho Pharmaceutical (Japan), which was approved by Japan’s MHLW in 2022 for treatment of advanced GIST after the tumors have progressed and no longer respond to tyrosine kinase kit inhibitors, we would like to refer to two previous excellent reviewer articles [17,18]. Patients received Pimitespib once daily, orally and on an empty stomach, for five consecutive days and a 2-day rest, in 21-day cycles with the starting dose of 160 mg/day, followed by dose reductions of three doses of 120, 80, and 40 mg/day as determined. Pimitespib-related AEs include severe diarrhea, eye disorders, and hemorrhage, which are similar to those reported for previous clinical trials with IV injections of ATP-binding inhibitors of Hsp90. The median progression-free survival was 4.2 months (95% confidence interval [CI] 1.9–6.2), the overall response rate was 0% (95% CI 0–16.1), and the disease control rate was 66.7% (95% CI 43.0–85.4) [19]. We argue that Pimitespib’s reduced systemic toxicity and detectable efficacy are due to its localized (oral) administration only to the GI tracks, so that it significantly avoids the main circulation system to affect other organs, and its (first) inhibition of tumor cell-secreted eHsp90α prior to entering tumor cells.

## 5. A Denial of Oversight: Both Intracellular and Extracellular Hsp90 Were Actually Targeted by Hsp90 ATP-Binding Inhibitors in All Previous Cancer Clinical Trials

**Figure 1:**
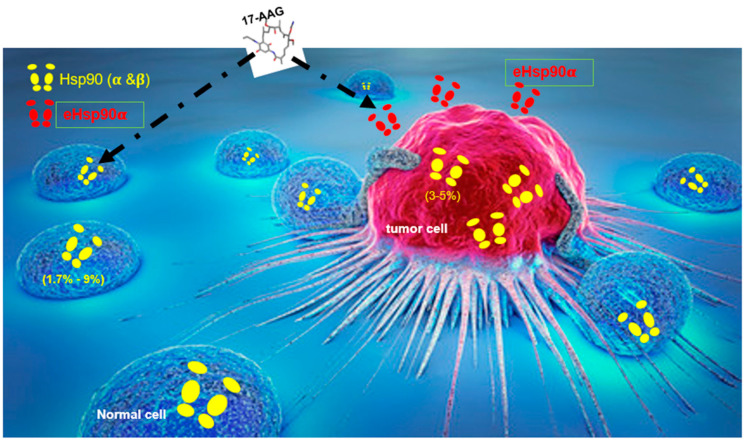
The ATP-binding inhibitors of Hsp90, such as 17-AAG, in fact blocked both intracellular and extracellular Hsp90β and Hsp90α in previous clinical trials. Studies since 2004 already showed that bead-bound or chemically modified membrane-impermeable forms of Hsp90 ATP-binding inhibitors, such as 17-AAG, inhibited tumor cell invasion in vitro and tumor metastasis in mice. Thus, 17-AAG and related ATP-binding inhibitors that had gone to clinical trials actually targeted both intracellular and extracellular Hsp90. The MOA of the inhibitors presented to FDA for clinical trial approvals was incorrect. Images taken from “Online Science Note for All” and modified.

The MOA (mechanism of action) of the ATP-binding Hsp90 inhibitors described in all previous IND applications to FDA for clinical trials was partially incorrect, if not misleading. As early as 2004, Eustace and colleagues demonstrated that geldanamycin (GA), the prototype ATP-binding Hsp90 inhibitor, could inhibit tumor cell invasion even when chemically tethered to beads, thereby preventing its entry into cells and its interaction with intracellular Hsp90 [20]. This was the first direct evidence that ATP-binding Hsp90 inhibitors exert their effects through simultaneous inhibition of both intracellular Hsp90 and extracellular Hsp90 (eHsp90). A schematic illustration of this new finding is shown in Figure 1. As illustrated, all the six classes of Hsp90 ATP-binding inhibitors, which have entered various cancer clinical trials, would bind and inhibit both intracellular (yellow) and extracellular (red) Hsp90 proteins. Whatever the reported outcomes of the clinical trials, they were actually balanced effects of both inhibitions. Remarkably, this potentially paradigm-shifting finding has been almost entirely overlooked, even by participants of the very original study, who have since continued to frame ATP-binding inhibitors exclusively in terms of intracellular targeting. Follow-up confirmation came from at least two closely related studies and a review article in 2013 by authors of this article, but again these studies too have been incomprehensively ignored within the Hsp90 research community [21,22,23]. First, a 2008 study by Tsutsumi and colleagues designed a cell-impermeable analog of 17-DMAG, DMAG-N-oxide, and conducted a series of carefully controlled experiments [22]. They reported the following:

1. DMAG-N-oxide binds Hsp90 with an affinity similar to that of 17-DMAG.
2. Both compounds inhibit the ATPase activity of purified Hsp90.
3. DMAG-N-oxide does not block tumor cell proliferation (likely since it did not get inside cells).
4. As expected, DMAG-N-oxide does not induce degradation of client proteins.
5. Unlike 17-DMAG, DMAG-N-oxide does not alter the total cellular Hsp90 levels.
6. Binding of DMAG-N-oxide to eHsp90 does not change its half-life.
7. DMAG-N-oxide inhibits invasion of multiple tumor cell types in vitro and suppresses B16 tumor cell lung colonization in a mouse metastasis model.

Although 17-DMAG showed greater inhibition of invasion in dose–response assays in vitro than DMAG-N-oxide, its stronger effect likely resulted from a combination of intracellular toxicity (such as by targeting Hsp90β), inhibition of extracellular Hsp90α (eHsp90α), and/or the inherent structural instability of DMAG-N-oxide. The finding that DMAG-N-oxide does not inhibit tumor cell proliferation is especially noteworthy, suggesting that eHsp90 is dispensable for basic, non-neoplastic processes such as cell division. Second, a 2014 study by Jay’s group reported similar results with STA-12-7191, a cell-impermeable analog of ganetespib. Despite some degree of unintended cellular uptake, STA-12-7191, like GA-beads and DMAG-N-oxide, bound eHsp90 and inhibited tumor cell migration in vitro [21]. These results are consistent with those from a study from Li’s group that utilized CRISPR–Cas9 to knockout Hsp90α in MDA-MB-231 breast cancer cells. Zou et al. showed that the knockout cells proliferated normally but completely lost the ability to invade in vitro or to form tumors in mice [24]. As previously demonstrated, normal cells do not secrete Hsp90 under physiological conditions. eHsp90α is only detected in conditioned medium under stress, such as hypoxia or tissue damage, e.g., wound healing, or pathological states such as tumorigenesis [25]. This explains why cell-impermeable Hsp90 inhibitors show minimal toxicity to the fundamental cellular processes. Supporting this conclusion, three monoclonal antibodies targeting different regions of Hsp90—1G6-D7 (linker region), 4c5 (N-terminal), and SPA-830 (C-terminal region)—have each been shown to strongly block eHsp90-driven migration of both normal and cancer cells, as well as tumor formation and metastasis in mouse models [22,24,26]. To remind the Hsp90 community, Li and colleagues published a review article entitled “Extracellular Hsp90 (eHsp90) as the actual target in clinical trials: intentionally or unintentionally” in 2013 [23]. Again, few have paid an attention to the “warning” over the past 10 years and everyone remains religiously committed to the doctrine that Hsp90 ATP-binding inhibitors target intracellular Hsp90 chaperones only.

## 6. Hsp90β: The Origin of Toxicity and Limit of Maximum Tolerated Dose (MTD) of Hsp90 Inhibitors

**Figure 2:**
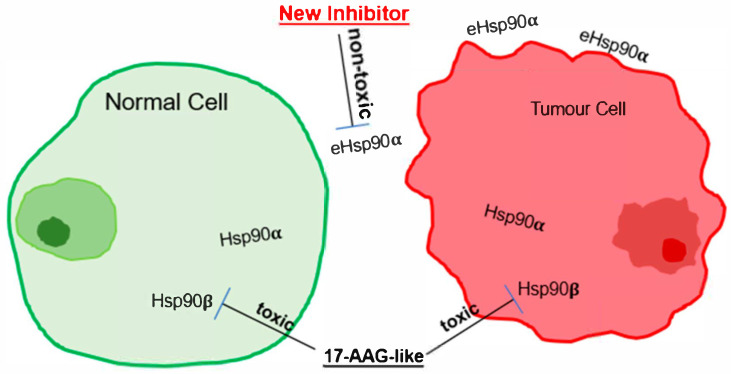
Targeting the non-chaperone functions of eHsp90α is safer and more effective. As schematically illustrated, ATP-binding inhibitors such as 17-AAG cause cellular toxicity in vitro and possibly the reported lines of toxicity in clinical trials by inhibiting the life-supporting role of intracellular Hsp90β. Overwhelming evidence shows that eHsp90α is an alternative, safer, and effective target for a new generation of anti-cancer therapeutics.

Higher toxicity than efficacy is the leading cause for cancer therapeutic candidates to fail in clinical trials. Without an exception, this common blockade has prevented each of the two dozen Hsp90 ATP-binding inhibitors tested in clinical trials to date from gaining FDA approval. Accumulating evidence over the past two decades strongly suggests that inhibition of the constitutively and ubiquitously expressed Hsp90β—not the inducible and variably expressed Hsp90α—is the principal cause of dose-limiting toxicity (DLT) in humans. Gene knockout studies in both cultured cells and mouse models have unequivocally demonstrated that Hsp90β is essential for cellular and organismal survival, whereas Hsp90α is dispensable in both contexts. To explain the distinct biological roles of Hsp90α and Hsp90β, we have previously proposed the “nut and shell” theory, in which Hsp90β is the core—essential for life—whereas Hsp90α serves as a guard, shielding Hsp90β from cell-penetrated inhibitors. ATP-binding Hsp90 inhibitors bind indiscriminately to the ATP-binding pockets of both Hsp90 isoforms; however, it is the inhibition of Hsp90β’s essential housekeeping functions that most likely cause clinical toxicity. By contrast, Hsp90α acts as a first-line “shield” or “buffer,” titrating the effect of penetrated inhibitors and thereby mitigating the level of damage to Hsp90β. Because Hsp90β expression is relatively constant across cell types and tissues in both mice and humans, the degree of protection afforded by Hsp90α explains the highly variable toxicities in different organs. Higher Hsp90α levels would more effectively buffer Hsp90β, reducing toxicity; conversely, lower Hsp90α levels would leave Hsp90β more vulnerable to cell-penetrated inhibitors. McGready et al. reported that the relatively higher cellular toxicity of STA-12-7191 was due to “leakage” during compound breakdown, generating small amounts of a membrane-permeable byproduct capable of entering cells [20]. Similarly, even the membrane-impermeable DMAG-N-oxide was reported to produce a metabolic byproduct that caused retinal toxicity—likely by penetrating cells and inhibiting Hsp90β. In contrast, Hsp90α is dispensable for normal development and homeostasis in mice, with the only notable exception being impaired spermatogenesis in homozygous knockout male mice. CRISPR–Cas9 deletion of Hsp90α in cell lines—including MDA-MB-231 breast cancer cells, mouse embryonic fibroblasts (MEFs), and others—had no effect on cell survival or proliferation [24,27]. To date, all studies support the notion that eHsp90α is an ideal, toxicity-free target for cancer therapeutics. We propose, as illustrated in Figure 2, that selectively targeting eHsp90α, but not via the N-terminal ATP-binding site of intracellular Hsp90β, may allow the highest MTD (maximum tolerated dose) with minimum toxicity. For detailed experimental support of this notion, readers are referred to two comprehensive review articles on Hsp90α inhibitors, covering its secretion mechanism, mode of action, supporting evidence from cellular and genetic studies, and clinical data from over 18 human studies [28,29].

Alternatively, Suhasini and colleagues raised an interesting concept of “epichaperome” and suggested that Hsp90, per se, is not a target in cancer. Instead Hsp90 may become a viable drug target only when it integrates into the epichaperome networks, providing a different explanation for the poor performance of the previous Hsp90 inhibitors in randomly selected patient populations [30].

## 7. Why Haven’t Membrane-Impermeable ATP-Binding Inhibitors or Anti-Hsp90 Antibodies Reached Clinical Trials?

Membrane-impermeable small molecules face multiple barriers to distribution in the human body, severely limiting their bioavailability. Antibodies, while highly specific, are costly to manufacture and have limited tissue penetration in vivo. However, as previously reported, even membrane-impermeable analogs such as DMAG-N-oxide could produce membrane-permeable metabolic byproducts capable of entering cells and causing unintended-target toxicity, including retinal damage. Therefore, targeting the ATP-binding site of intracellular Hsp90—especially Hsp90β—either deliberately or inadvertently, could lead to a therapeutic dead end. A deeper problem, however, might be a conceptual one: the Hsp90 community remains so entrenched in outdated, loosely supported mechanistic assumptions, while largely ignoring potentially paradigm-shifting findings in recent years.

## 8. An Arriving New Hope for Anti-Hsp90 Therapeutics: Targeting the Non-Chaperone Epitope in eHsp90α

There have been two major mechanisms proposed for the mechanism of action by eHsp90α. A detailed discussion of this debate can be found in the review article [29].

**(1) Chaperone model**—eHsp90α functions outside cells as an ATP-dependent chaperone, as its intracellular counterpart does. The center of this model is that eHsp90α chaperones and activates MMP2 to promote tumor cell invasion. In fact, no direct evidence supports a requirement for MMP2 or other MMPs in eHsp90α-stimulated tumor cell migration or invasion. Cheng et al. reported that MMP inhibitors had little effect on purified recombinant Hsp90α-driven cell migration in vitro. Using various ATPase-defective Hsp90α mutant (E47A, E47D, D93N) proteins, Cheng and colleagues demonstrated that extracellular functions of Hsp90α is completely independent of ATPase activity [31]. In fact, the entire pro-motility and pro-survival functions of eHsp90α reside in a 115 amino acid fragment, called F-5, between the charged and the middle domains [24,32].

**(2) The Non-chaperone “eHsp90α > LRP-1 receptor signaling” model**—The non-chaperone mechanism centers on the so-called “eHsp90α > LRP1 receptor signaling axis”, first characterized by Li’s laboratory [24,33]. Using deletion mutagenesis, they identified a 115–amino acid (236–350) region, F-5, between the linker (LR) and middle (M) domains as both necessary and sufficient for full-length eHsp90α’s pro-survival, pro-motility, and pro-invasion effects in vitro and in vivo [32,34,35]. Recombinant F-5 peptide alone promotes skin cell migration and accelerates wound healing in mice and pigs, equivalent to the full-length Hsp90α protein. Mechanistically, F-5 binds to the subdomain II of the extracellular region of low-density lipoprotein receptor-related protein 1 (LRP1), signaling via the NPVY motif in its cytoplasmic tail of LRP-1 and inducing serine-473 (but not threonine-308) phosphorylation in Akt1/2 kinases. Activated Akt kinases led to cell migration [33]. A recent study showed that the function of eHsp90α is even evolutionarily conserved in yeast fungus, *C. albicans*, to support its virulence [36].

## 9. Therapeutic Targeting of the F-5 Region of eHsp90α

A critical step in drug development is to achieve an effective MTD, initially estimated in animal models as the highest single or short-term dose that does not cause mortality, significant body weight loss, or other signs of acute toxicity unrelated to carcinogenicity. In humans, the MTD is refined during phase I trials by escalating doses across patient cohorts until the highest dose with acceptable side effects is identified [37]. While body weight suppression was once the primary benchmark, modern safety assessment also incorporates physiologic, pharmacologic, and metabolic biomarkers as more sensitive indicators of systemic stress [38]. As a result, the overall success rate for oncology drugs remains below 5% [39]. For the vast majority of the >1000 FDA-approved anti-cancer drugs administered intravenously, dose-limiting toxicity to normal tissues constrains elevation of MTD, reducing therapeutic effectiveness. In many cases of cancer drug targets in the past, the therapeutic window is too narrow to achieve meaningful tumor suppression without unacceptable damage to healthy tissue—an outcome reminiscent of the ancient Chinese military maxim: 杀敌一千, 自失八百 meaning “killing one thousand enemy soldiers, but losing eight hundred of your own” in translation, which is regarded as 得不偿失 (the cost outweighs the benefit) or a pyrrhic victory. This raises a pivotal question: is there a cancer drug target critical for tumor progression but largely dispensable for normal homeostasis? For the first time, eHsp90α appears to fit this profile.

Unlike previous (conventional) oncogenic targets, the following points can be made:

1. **Selective secretion:** eHsp90α is secreted only under stress by normal cells—primarily for wound repair—yet is constitutively secreted by many cancer cells.
2. **Non-essential for life**: Hsp90α, whether intracellular or extracellular, is not essential for survival.
3. **Clinically elevated:** The eHsp90α level is found elevated in the plasma of patients with almost all types of cancer in humans.
4. **Pro-metastatic threshold:** Plasma concentrations >100 ng/mL closely correlate with the stages of tumor metastasis.
5. **Unique functional epitope:** The entire activity of eHsp90α is located in the F-5 region.

**Figure 3:**
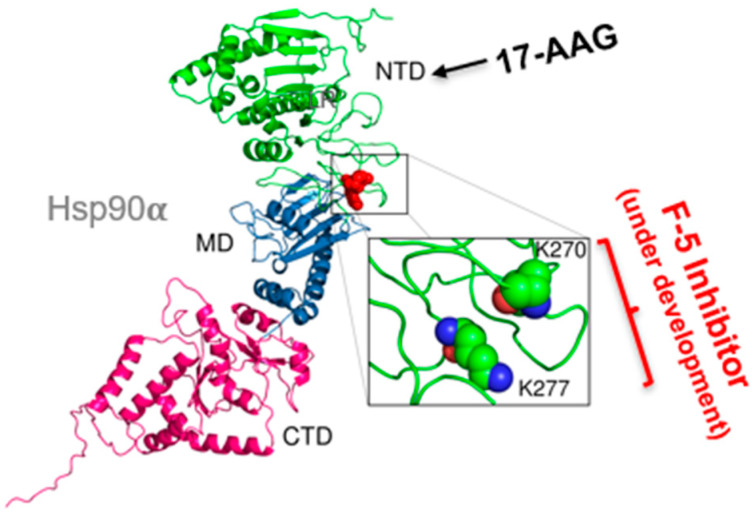
Targeting the dual lysine residues in the F-5 region of eHsp90α. The 115 amino acid (a.a.236–a.a.350) fragment, termed fragment-5 (F-5), between the charged region and middle domain contains the full function of the 732 amino acid full-length eHsp90α in vitro and in vivo [32]. The lysine residues, K-270 and K277, are the “active site” of F-5 [24]. As illustrated, while targeting the ATP-binding site has failed in more than 180 clinical trials, inhibitors targeting the dual lysine site in F-5 are currently being developed.

Within the F-5 region, Zou and colleagues pinpointed a dual lysine motif (Lys-270/Lys-277), conserved in all Hsp90α isoforms but absent from all Hsp90β isoforms from yeast to man. Mutating either lysine abolished Hsp90α’s pro-motility and tumor-promoting functions; conversely, introducing these two lysine residues into Hsp90β conferred Hsp90α-like activity [24]. A monoclonal antibody, 1G6-D7, generated against KLH-conjugated F-5 binds near the dual lysine motif and shows the strongest inhibition of both recombinant Hsp90α-driven migration, constitutive motility of tumor cells, and tumor formation in mice. These findings conclusively show that neither the N-terminal ATPase domain nor the C-terminal dimerization/cofactor-binding domain is required for eHsp90α’s extracellular functions. Instead, the dual lysine motif within F-5 represents a highly specific, non-toxic target for next-generation Hsp90 therapeutics. As illustrated in Figure 3, the dual lysines reside in a highly charged surface region, making it a new molecular “bait” for screening small molecules that selectively bind and inhibit tumor-secreted eHsp90α. In theory, such “dual lysine” inhibitors would block the ATPase-independent extracellular signaling of eHsp90α without interfering with the ATP-dependent intracellular chaperone function of Hsp90, thus greatly reducing systemic toxicity in the patients. Most significantly, this selectivity could enable higher and effective MTD, maximizing suppression of tumor invasion and metastasis while minimizing harm to normal tissues.

## 10. Inhibition of eHsp90α May Not Be Recommended Immediately After Surgery

Evidence from Hsp90α-knockout mice demonstrates significant delay in wound healing, and this delay could be rescued by topical supplementation of recombinant eHsp90α protein [40]. This study provides direct proof that eHsp90α plays a driver’s role in the wound repair process. Supporting this conclusion, topical application of recombinant Hsp90α or its F-5 fragment accelerates closure of excision and burn wounds, reduces the delay in diabetic wound healing in mice from 45 to 18 days [29], and promotes wound closure in both normal and diabetic wounds in pig models [41]. Therefore, eHsp90α protein itself is a promising therapeutic for enhancing wound repair. Because wound healing and tumorigenesis share striking similarities in their microenvironmental conditions [42] and biological hallmarks [43], caution is warranted in the clinical context. Cancer patients should avoid simultaneous use of both anti-eHsp90α agents and wound healing-promoting eHsp90α therapeutics, and the same caution applies to patients recovering from major injuries. In particular, while next-generation anti-eHsp90α therapeutics may offer markedly reduced systemic toxicity compared to currently available anti-cancer drugs, they could still impair wound healing in patients, especially those with open surgical wounds. Therefore, inhibition of eHsp90α immediately after surgery is not recommended until the wound has been declared clinically closed.

## 11. Conclusions

Since 1999, none of the ~180 mono- or combination-therapy cancer clinical trials targeting the intracellular Hsp90 chaperone machinery has received FDA approval for cancer treatment in humans. The initial MOA that received the FDA approvals of the Hsp90 inhibitor clinical trials is now called into question, since these inhibitors actually blocked both intracellular and extracellular Hsp90 and extracellular Hsp90α supports tumor formation and metastasis. The widely accepted “Hsp90 inhibitor binding–client protein degradation” model does not apply to many types of tumor cells. The pronounced variation in Hsp90 expression across human organs, with levels being low or undetectable in tissues such as the eye, muscle, and heart, makes it impossible to establish an effective MTD that selectively damages tumors without (more) harming normal tissues. The next generation of therapeutics should target tumor-secreted eHsp90α, which has demonstrated both low toxicity and high efficacy in animal models.

## Data Availability

No new data were created or analyzed in this study.

## Abbreviations

The following abbreviations are used in this manuscript:

17AAG	17-N-allylamino-17-demethoxygeldanamycin
17-DMAG	17-dimethylaminoethylamino-17-demethoxygeldanamycin
MMP2	matrix metalloproteinase-2
IND	investigational new drug
MOA	mechanism of action
eHsp90	Extracellular Hsp90
TAS-116	Pimitespib
Co-IP	co-immunoprecipitation
MS	mass spectrometry
GA	geldanamycin
DLT	dose-limiting toxicity
MEF	mouse embryonic fibroblasts
MTD	maximum tolerated dose
AE	adversary effect
LRP1	Lipoprotein receptor-related protein 1
